# Ultrasound image-based deep learning to differentiate tubal-ovarian abscess from ovarian endometriosis cyst

**DOI:** 10.3389/fphys.2023.1101810

**Published:** 2023-02-07

**Authors:** Ping Hu, Yanjuan Gao, Yiqian Zhang, Kui Sun

**Affiliations:** ^1^ Department of Ultrasound, The First People’s Hospital of Datong, Datong, China; ^2^ Department of Women’s Healthcare Rehabilitation, Boai Hospital of Zhongshan Affiliated to Southern Medical University, Zhongshan, China; ^3^ Department of Radiology, Shandong Provincial Hospital Affiliated to Shandong First Medical University, Jinan, China

**Keywords:** artificial intelligence, deep learning, convolutional neural networks, tubal-ovarian abscess, ovarian endometriosis cyst

## Abstract

**Objectives:** We developed ultrasound (US) image-based convolutional neural networks (CNNs) to distinguish between tubal-ovarian abscess (TOA) and ovarian endometriosis cyst (OEC).

**Methods:** A total of 202 patients who underwent US scanning and confirmed tubal-ovarian abscess or ovarian endometriosis cyst by pathology were enrolled in retrospective research, in which 171 patients (from January 2014 to September 2021) were considered the primary cohort (training, validation, and internal test sets) and 31 patients (from September 2021 to December 2021) were considered the independent test cohort. There were 68 tubal-ovarian abscesses and 89 OEC, 4 TOA and 10 OEC, and 10 TOA and 21 OEC patients belonging to training and validation sets, internal sets, and independent test sets, respectively. For the model to gain better generalization, we applied the geometric image and color transformations to augment the dataset, including center crop, random rotation, and random horizontal flip*.* Three convolutional neural networks, namely, ResNet-152, DenseNet-161, and EfficientNet-B7 were applied to differentiate tubal-ovarian abscess from ovarian endometriosis cyst, and their performance was compared with three US physicians and a clinical indicator of carbohydrate antigen 125 (CA125) on the independent test set. The area under the receiver operating characteristic curves (AUROCs) of accuracy, sensitivity, and specificity were used to evaluate the performance.

**Results:** Among the three convolutional neural networks, the performance of ResNet-152 was the highest, with AUROCs of 0.986 (0.954–1). The AUROCs of the three physicians were 0.781 (0.620–0.942), 0.738 (0.629–848), and 0.683 (0.501–0.865), respectively. The clinical indicator CA125 achieved only 0.564 (0.315–0.813).

**Conclusion:** We demonstrated that the CNN model based on the US image could discriminate tubal-ovarian abscess and ovarian endometriosis cyst better than US physicians and CA125. This method can provide a valuable predictive reference for physicians to screen tubal-ovarian abscesses and ovarian endometriosis cysts in time.

## 1 Introduction

Annually, pelvic inflammatory disease (PID) accounts for over 700,000 cases in the United States (Gradison, 2012). A tubal-ovarian abscess (TOA) is a complex, severe, acute-onset complication of PID caused by the infection of the female upper genital tract, found in 15%–34% of patients (Mohammad et al., 2022). Delayed diagnosis contributes to inflammatory sequelae, including infertility, ectopic pregnancy, and chronic pelvic pain (Curry et al., 2019). If the abscess ruptures, it can cause more acute and severe complications such as acute diffuse peritonitis or sepsis, which can be life-threatening (Brunham et al., 2015). Ovarian endometriosis cyst (OEC) is the main presence of endometrial-type mucosa and stroma outside the uterine cavity (Vercellini et al., 2014). It is primarily ectopic to the ovary and is a chronic inflammatory disease dependent on estrogen, affecting women in their reproductive years and producing clinical symptoms similar to TOA. In contrast to TOA, its progression is relatively slow, and the prognostic risk is much lower than that of TOA. Therefore, early differential diagnosis and timely intervention treatment play a crucial role in patients’ overall course of treatment.

Ultrasound (US) is often regarded as the first-line imaging examination method due to its many advantages, such as low cost and non-invasiveness. The atypical sonographic appearance of TOA is often confused with other cystic masses, especially OEC. Because both of them frequently occur in the ovary and its surrounding fallopian tubes, they are cystic masses with poor sound transmission (absence of color Doppler flux within the cyst) and adhesion to the surrounding tissues. Another report showed that TOA originating from non-gynecological diseases might remain challenging to differentiate from OEC on computed tomography (CT) (Revzin et al., 2016). Therefore, this problem has caused a dilemma for clinicians to some extent.

Convolutional neural networks (CNNs) are frequently used for imaging tasks in deep learning. By combining different numbers of transformation methods (data augmentation and attention mechanism), CNNs’ learning ability can be enhanced and used to solve practical problems well (LeCun et al., 2015). It has been widely reported that CNNs have been applied for classification tasks in medical images (Anwar et al., 2018; Liang et al., 2020; Che et al., 2021). Liang et al. (2020) showed that using segmented ultrasound images can improve the performance of CNN models for the diagnosis of breast and thyroid nodules. Moreover, CNN not only can be applied to medical images (Rahaman et al., 2020) but also performs well in histopathology images (Chen et al., 2022b; Chen et al., 2022c; Li et al., 2022), microorganism images (Zhang et al., 2021; Zhao et al., 2022), and cell image and video analysis (Chen et al., 2022a; Liu et al., 2022). Therefore, we aim to determine whether the CNN models can precisely differentiate TOA from OEC on US images to assist physicians in making decisions in the clinic.

## 2 Materials and methods

### 2.1 Subjects and datasets

**FIGURE 1:**
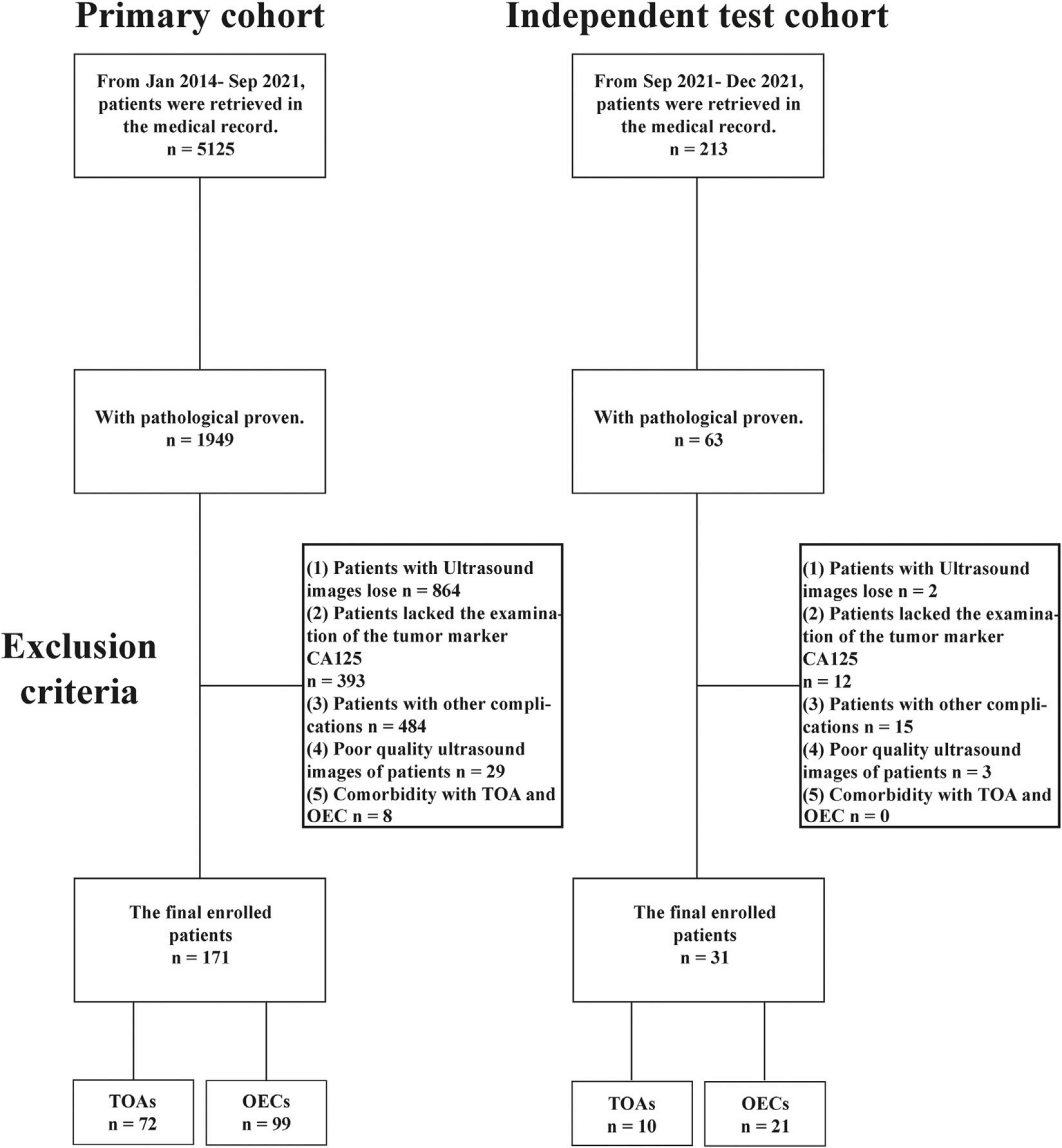
Inclusion workflow of subjects in this study.

The Institutional Review Board approved this retrospective study, and the requirement to obtain informed consent was waived. We retrieved the pathological database from January 2014 to September 2021 in our institution. The patients with histologically confirmed TOA or OEC were enrolled in our study as a primary cohort. A total of 157 patients were involved in the training and validation sets, and 14 patients were involved in the internal test set. In addition, from September 2021 to December 2021, we included 31 patients as an independent test cohort. Figure 1 summarizes the selection process of the patient cohort in the current study. The basic characteristics of the patients, including age, weight, lesion diameter, tumor marker, biochemical examination, and US diagnosis reports, were collected from the medical record. To ensure that the CNN models could learn better feature information from the images, three or four of the most typical US images of each patient were selected by an ultrasound physician with more than 10 years of experience for further analysis.

### 2.2 US image data acquisition protocol

**FIGURE 2:**
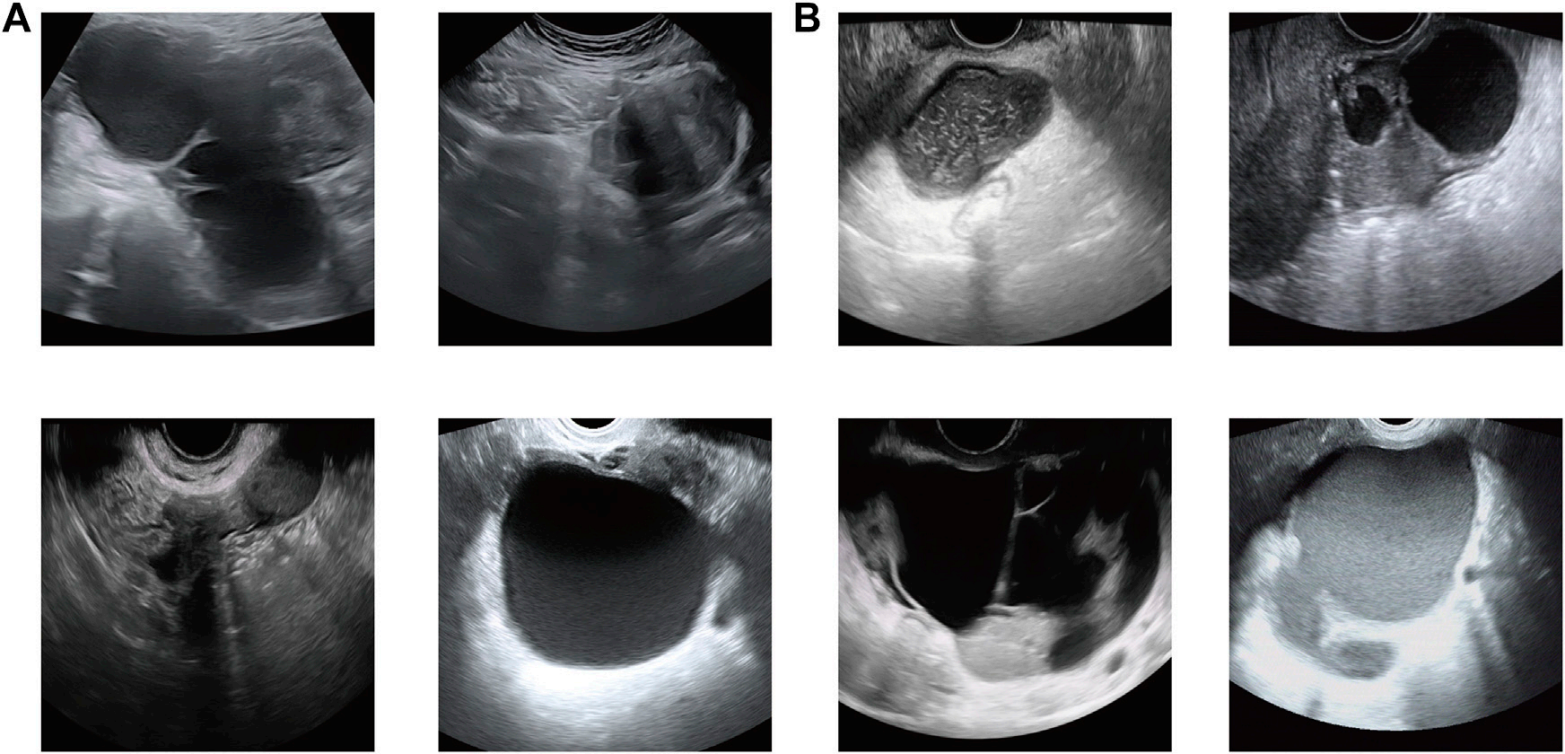
Sample dataset image in this study. **(A)** Ovarian endometriosis cyst (OEC) and **(B)** tubal-ovarian abscess (TOA).

All subjects were positioned in the lithotomy position with the vulva exposed for continuous multi-section scanning of the uterus, cervix, and ovary using an ACUSON Sequoia system (Siemens Healthineers, Germany) or LOGIQ (GE Healthcare, United States) ultrasonic diagnostic system. All equipment has the default gynecological examination mode, equipped with 3.5–6.0 MHz convex array probes and 7.5–12 MHz intracavitary (transvaginal) probes. These examinations were performed by three ultrasonologists with at least 5 years of clinical experience. The US images were saved in the Joint Photographic Experts Group (JPEG) format in the picture archiving and communication system (PACS) workstation. Figure 2 shows the sample dataset image in this study.

### 2.3 Data pre-processing

Data augmentation is the most common strategy for a limited dataset (Wang et al., 2021), which can increase the dataset up to ten times its original scale *via* random geometric image and color transformations. Our study used a package named “Torchvision” to perform data augmentation, including center crop, random rotation, random horizontal flip, random vertical flip, and random color jitter (brightness, contrast, saturation, and hue). On the one hand, this strategy can ensure that the model pays more attention to the lesions rather than the noise information. On the other hand, the data augmentation method can also help avoid network overfitting and learn more details of the image on the training dataset (Kayalibay et al., 2017). We resized the image to 224 × 224 pixels to standardize the distance scale and meet the input requirement of the model.

### 2.4 Deep learning model construction, training, and validation

Nowadays, CNNs are the most popular type of deep learning frameworks for medical image analysis (Litjens et al., 2017). There is also no doubt over the remarkable performance delivered by CNNs in the classification tasks of the medical imaging field. The classical CNN models included AlexNet (Krizhevsky et al., 2017), VGG-16/19Net (Simonyan and Zisserman, 2014), and residual neural network (ResNet) (He et al., 2016).

In this research, three CNNs were pre-trained *via* the ImageNet natural image library (http://www.imagenet.org/) and were used to deploy and differentiate TOA from OEC based on US images, including ResNet-152, DenseNet-161, and EfficientNet-B7.

The weights of the model learned from natural images may not be directly suitable for medical images. Therefore, we used transfer learning for the analysis of ultrasound images. CNNs with pre-trained weights were used to fine-tune our training and validation sets. The best parameters of the CNN models were selected *via* the highest accuracy on the validation set. The stochastic gradient descent (SGD) optimizer with 0.9 momentum trained the network. The batch sizes were 166, the epochs were 300, and the learning rate was 0.01. It was always a better choice for the classification task to use the cross-entropy function as the loss function. The use of batch normalization has many advantages. For example, 1) it can accelerate the training speed of the network, solving the problem of gradient vanishing and 2) it can improve the network’s generalization ability, reducing the overfitting phenomenon but not relying on regularization methods such as dropout/L2. We changed the last fully connected layer of the network from 1,000 to 2 to accommodate our task.

Internal and independent test sets were used to assess the generalization of the CNNs. The output of the CNN models was regarded as predicted probability, and we chose the class of the highest probability as the prediction outcome. As mentioned previously, there were multiple images from each patient which could cause an inaccurate overcount of the sample size and would falsely decrease the estimated uncertainty. In order to remove this phenomenon, a common way is to take the average output prediction from images as an individual probability, which produces a more robust estimation. We also used the clinical indicator CA125 for further comparison. Moreover, we invited three ultrasound physicians with more than 10 years of experience to implement a reader test. The performance of the reader test, CA125, and CNN models was compared on an independent test set.

### 2.5 Statistical analysis

The area under the receiver operating characteristic curves (AUROCs), accuracy, sensitivity, specificity, true-positive rate (TPR), false-positive rate (FPR), true-negative rate (TNR), false-negative rate (FNR), positive predictive value (PPV), negative predictive value (NPV), and F1-score were used to evaluate the performance of the CNN models, sonographers, and CA125. The comparison of different AUROCs was carried out using the DeLong test (DeLong et al., 1988), and *p* < 0.05 was considered a significant statistical difference. The Shapiro–Wilk test was used to evaluate continuous data distribution. If the data presented normal distribution, the *t*-test was used; otherwise, the Mann–Whitney *U*-test was used. The data with normal distribution were indicated by the mean ± SD, and the data with non-normal distribution were indicated by the median (IQR). The statistical analysis was conducted using R software version 4.1.2 (https://www.r-project.org/). The construction, training, and testing of the CNN models were carried out under the PyTorch framework version 1.9.0 (https://pytorch.org/) of Python version 3.8 (http://www.python.org/). The computer core hardware used in this study includes a CPU with Intel Core 12th Gen i9-12900K, a GPU with Nvidia RTX 3060 Ti 8 GB, and a RAM with Kingston DDR4-3600 64 GB.

## 3 Results

### 3.1 Patient characteristics

A total of 202 patients were enrolled in this study, of which 171 patients belonged to the primary cohort (training, validation, and internal test sets) while 31 patients (TOA patients, *n* = 10; OEC patients, *n* = 21) belonged to the independent test cohort. The training and validation sets included 68 TOA and 89 OEC patients, and the internal test set included 4 TOA and 10 OEC patients. We obtained 456 images from the training and validation sets, 43 from the internal test set, and 94 from the independent test set. The median age was 40 (33, 47) years (range, 20–60 years) for the training and validation sets, the mean age was 41.79 ± (6.92) years (range, 28–51 years) for the internal test set, and the median age was 38 (27, 44) years (range, 20–48 years) for the independent test set. The details of the patient characteristics are given in Table 1.

**TABLE 1:** Baseline characteristics of the subjects in this study.

Baseline characteristic	Training and validation sets		Internal test set		Independent test set	
	TOA patients (*n* = 68)	OEC patients (*n* = 89)	TOA patients (*n* = 4)	OEC patients (*n* = 10)	TOA patients (*n* = 10)	OEC patients (*n* = 21)
Age, years	45.00 (39.00, 49.25)	35.00 (30.00, 44.00)	48.75 ± 1.71	39.00 ± 6.16	42.00 (38.50, 45.75)	31.00 (26.00, 43.00)
Weight, kg	58.37 (55.00, 62.63)	60.00 (54.00, 66.50)	51.50 ± 10.47	59.85 ± 8.34	58.56 (56.00, 61.88)	57.00 (52.00, 61.00)
WBC, 10^9^/L	11.55 (7.65, 15.58)	5.90 (5.00, 6.90)	7.08 ± 1.73	5.64 ± 1.18	12.75 (9.78, 17.00)	6.10 (5.50, 7.20)
RBC, 10^12^/L	4.00 (3.80, 4.30)	4.30 (4.10., 4.60)	4.30 (3.98, 4.43)	4.40 (4.08, 4.40)	3.99 ± 0.40	4.29 ± 0.43
CA125, μ/mL	50.60 (24.55, 69.22)	57.8 (33.30, 98.30)	74.70 (9.88, 152.80)	80.40 (38.15, 85.00)	73.37 (28.98, 122.45)	49.20 (31.10, 61.22)
Largest diameters, cm	7.90 (6.45, 9.35)	6.00 (4.00, 7.30)	6.93 ± 2.19	6.80 ± 2.45	7.78 ± 3.02	6.97 ± 2.39

Abbreviation: TOA, tubal-ovarian abscess; OEC, ovarian endometriosis cyst; WBC, white blood cell; RBC, red blood cell; CA125, carbohydrate antigen 125.

### 3.2 Performance of three CNN models, ultrasound physicians, and CA125

Our model was trained on the training set with 2840 US images (355 original US images from the training set multiplied by the eight types of the data augmentation strategy). We first verified the predictive ability of the different CNN models on the internal test set, and ResNet-152 achieved an ideal performance to distinguish TOA and OEC patients, with AUROCs of 1 (95% CI: 1–1), an accuracy of 1 (0.785–1), a sensitivity of 1 (0.510–1), and a specificity of 1 (0.722–1). DenseNet-162 achieved diagnostic performance with AUROCs of 0.975 (0.906–1), an accuracy of 0.929 (0.685–0.996), a sensitivity of 1 (0.510–1), and a specificity of 0.9 (0.596–0.995). EfficientNet-B7 achieved diagnostic performance with AUROCs of 0.925 (0.760–1), an accuracy of 0.929 (0.685–0.996), a sensitivity of 0.750 (0.301–0.987), and a specificity of 1 (0.772–1).

Second, we verified the performance of different CNN models on the independent test set. For ResNet-152, the AUROCs, accuracy, sensitivity, and specificity were 0.986 (95% CI: 0.954–1), 0.968 (0.838–0.998), 0.9 (0.596–0.995), and 1 (0.845–1), respectively. For DenseNet-161, the AUROCs, accuracy, sensitivity, and specificity were 0.924 (0.791–1), 0.903 (0.751–0.967), 0.9 (0.596–0.995), and 0.905 (0.711–0.973), respectively. For EfficientNet-B7, the AUROCs, accuracy, sensitivity, and specificity were 0.976 (0.935–1), 0.936 (0.793–0.982), 1 (0.890–1), and 0.905 (0.711–0.973), respectively.

Obviously, the efficacy of the CNN models was superior to the diagnosis performed by sonographers and CA125. As shown in Table 2, on the independent test set, the AUROCs, accuracy, sensitivity, specificity, TPR, FPR, TNR, and FNR were only 0.781 (95% CI: 0.620–0.942), 0.774 (0.602–0.886), 0.8 (0.49–0.943), 0.762 (0.549–0.894), 0.8, 0.238, 0.762, and 0.2 for Reader 1; 0.738 (0.629–848), 0.645 (0.469–789), 1.00 (0.722–1), 0.476 (0.283–0.676), 1, 0.524, 0.476, and 0 for Reader 2; and 0.683 (0.501–0.865), 0.677 (0.501–0.814), 0.7 (0.397–0.892), 0.667 (0.454–0.828), 0.7, 0.333, 0.667, and 0.3 for Reader 3, respectively. Furthermore, in this study, the diagnostic efficacy of ResNet-152 was the highest among the three models.

**TABLE 2:** Performance of ResNet-152, DenseNet-161, EfficientNet-B7, CA125, and readers.

Evaluator	AUROC (95% CI)	Acc (95% CI)	Sen (95% CI)	Spe (95% CI)	NPV	PPV	FPR	TPR	TNR	FNR	F1-score
Internal test set											
ResNet-152	1.000 (1–1)	1 (0.785–1)	1 (0.510–1)	1 (0.722–1)	1	1	0	1	1	0	1
DenseNet-161	0.975 (0.906–1)	0.929 (0.685–0.996)	1 (0.510–1)	0.9 (0.596–0.995)	1	0.8	0.1	1	0.9	0	0.889
EfficientNet-B7	0.925 (0.760–1)	0.929 (0.685–0.996)	0.750 (0.301–0.987)	1 (0.772–1)	0.910	1	0	0.75	1	0.25	0.857
Independent test set											
ResNet-152	0.986 (0.954–1)	0.968 (0.838–0.998)	0.9 (0.596–0.995)	1 (0.845–1)	0.955	1	0	0.9	1	0.1	0.947
DenseNet-161	0.924 (0.791–1)	0.903 (0.751–0.967)	0.9 (0.596–0.995)	0.905 (0.711–0.973)	0.950	0.818	0.095	0.9	0.905	0.1	0.857
EfficientNet-B7	0.976 (0.935–1)	0.936 (0.793–0.982)	1 (0.890–1)	0.905 (0.711–0.973)	1	0.833	0.095	1	0.905	0	0.909
Reader 1	0.781 (0.620–0.942)	0.774 (0.602–0.886)	0.8 (0.49–0.943)	0.762 (0.549–0.894)	0.889	0.615	0.238	0.8	0.762	0.2	0.696
Reader 2	0.738 (0.629–848)	0.645 (0.469–789)	1 (0.722–1)	0.476 (0.283–0.676)	1	0.476	0.524	1	0.476	0	0.645
Reader 3	0.683 (0.501–0.865)	0.677 (0.501–0.814)	0.7 (0.397–0.892)	0.667 (0.454–0.828)	0.824	0.5	0.333	0.7	0.667	0.3	0.583
CA125	0.564 (0.315–0.813)	0.677 (0.501–0.814)	0.6 (0.313–0.832)	0.714 (0.5–0.862)	0.789	0.5	0.286	0.6	0.714	0.4	0.545

Abbreviation: AUROC, area under the receiver operating characteristic curve; Acc, accuracy; Sen, sensitivity; Spe, specificity; NPV, negative predictive value; PPV, positive predictive value; FPR, false-positive rate; TPR, true-positive rate; TNR, true-negative rate; FNR, false-negative rate; 95% CI, 95% confidence interval.

**FIGURE 3:**
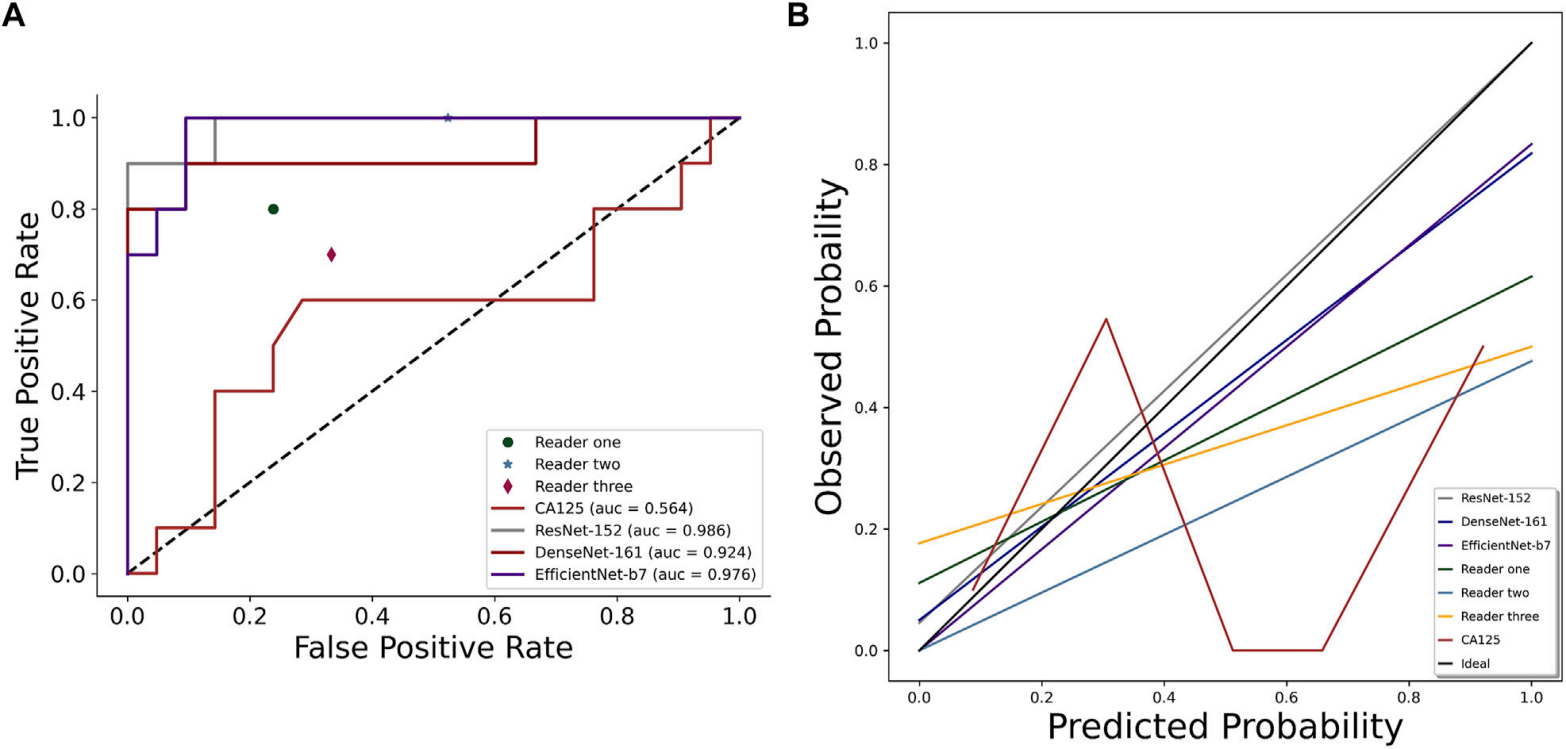
Comparison of AUROCs **(A)** and calibration curve **(B)** among CNN models (ResNet-152, DenseNet-161, and EfficientNet-B7), sonographer 1, sonographer 2, sonographer 3, and CA125 on the independent test set.

It is worth mentioning that the performance of the clinical predictor CA125 differs greatly from others. The AUROCs, accuracy, sensitivity, specificity, TPR, FPR, TNR, and FNR were only 0.564 (0.315–0.813), 0.677 (0.501–0.814), 0.6 (0.313–0.832), 0.714 (0.5–0.862), 0.6, 0.286, 0.714, and 0.4 for CA125, respectively. Figure 3A depicts the comparison of the AUROCs among different evaluators. According to the results verified on the independent test set, the AUROCs of ResNet-152 showed a significant difference compared with those of Reader 1 (*p* = 0.019), Reader 2 (*p* < 0.001), Reader 3 (*p* = 0.003), and CA125 (*p* = 0.003). There was no difference in the AUROCs of DenseNet-161 compared to Reader 1 (*p* = 0.184), but there was a difference in the AUROCs of Reader 2, Reader 3, and CA125 (*p* < 0.05). Compared to other evaluators, EfficientNet-B7 also showed differences (*p* < 0.05). The details are shown in Table 3. Figure 3B shows the consistency of predictive and observative probabilities among the CNN models, Reader 1, Reader 2, Reader 3, and CA125.

**TABLE 3:** Comparison of AUROC values by using the DeLong test among different evaluators on the independent test set.

Evaluator	AUROC value	*p*-value
ResNet-152 vs Reader 1	0.986 vs 0.781	0.019*
ResNet-152 vs Reader 2	0.986 vs 0.738	<0.001***
ResNet-152 vs Reader 3	0.986 vs 0.683	0.003**
ResNet-152 vs CA125	0.986 vs 0.564	0.003**
DenseNet-161 vs Reader 1	0.924 vs 0.781	0.184
DenseNet-161 vs Reader 2	0.924 vs 0.738	0.039*
DenseNet-161 vs Reader 3	0.924 vs 0.683	0.041*
DenseNet-161 vs CA125	0.924 vs 0.564	0.016*
EfficientNet-B7 vs Reader 1	0.976 vs 0.781	0.027*
EfficientNet-B7 vs Reader 2	0.976 vs 0.738	<0.001***
EfficientNet-B7 vs Reader 3	0.976 vs 0.683	0.004**
EfficientNet-B7 vs CA125	0.976 vs 0.564	0.003**
Reader 1 vs CA125	0.781 vs 0.564	0.158
Reader 2 vs CA125	0.738 vs 0.564	0.217
Reader 3 vs CA125	0.683 vs 0.564	0.452
Reader 1 vs Reader 2	0.781 vs 0.738	0.608
Reader 1 vs Reader 3	0.781 vs 0.683	0.157
Reader 2 vs Reader 3	0.738 vs 0.683	0.534

Abbreviation: AUROC, area under the receiver operating characteristic curve; * <0.05, ** <0.01, and *** <0.001.

**FIGURE 4:**
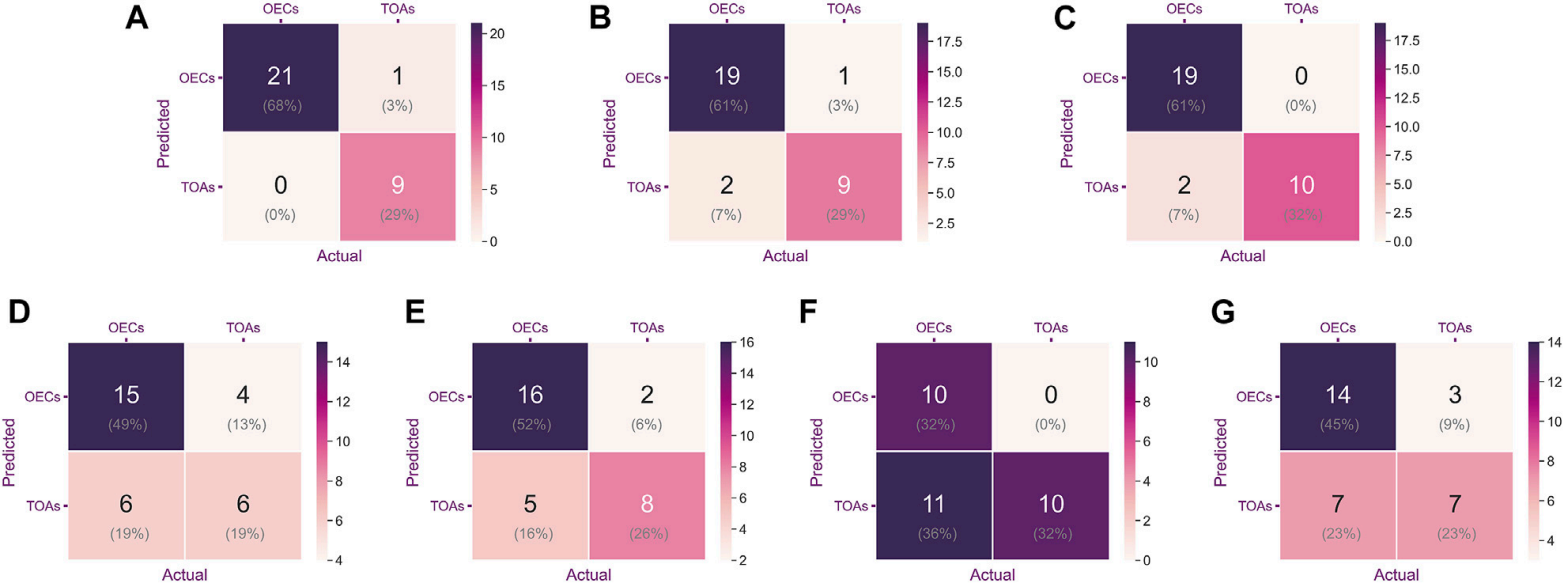
Confusion matrix was used to evaluate the classification accuracy of a model or indicator. The upper left and lower right corners represent the number of accurate predictions. From left to right are ResNet-152 **(A)**, DenseNet-161 **(B)**, EfficientNet-B7 **(C)**, CA125 **(D)**, Reader 1 **(E)**, Reader 2 **(F)**, and Reader 3 **(G)**.

**FIGURE 5:**
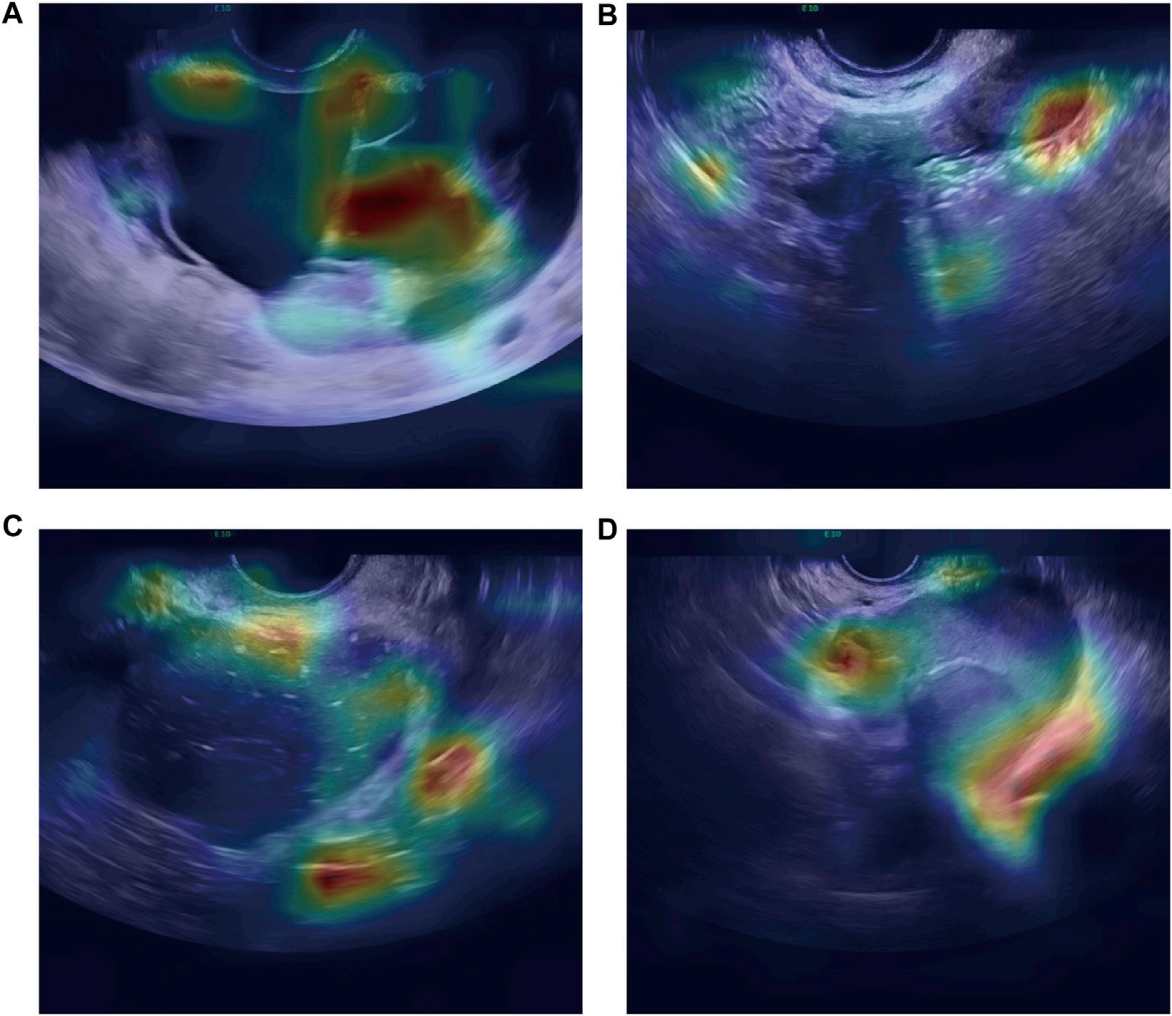
Heatmap illustrating the region of interest of the CNN model on OEC class **(A,B)** and TOA class **(C,D)**.

As shown in Figure 4, the confusion matrices could intuitively enable us to understand the diagnostic performance of the CNN models, ultrasound physicians, and clinical predictor CA125. Gradient-weighted class activation mapping (Grad-CAM) could be even better at telling us what the CNN models focused on and how the CNN models identified the slight difference between TOA and OEC. Figure 5 depicts the heatmap generated by Grad-CAM; the red area represents highly predictive, and the blue area represents low predictability. The deeper the color is, the higher will be the probability of being the discriminative region.

## 4 Discussion

We proposed CNN-based models for automatically classifying TOA and OEC patients in this retrospective study. These CNN models achieve excellent diagnostic performance in both internal and independent test sets, regardless of whether it is ResNet-152, DenseNet-161, or EfficientNet-B7. Based on our research, it is revealed that such models can be useful in identifying TOA and OEC. If TOA can be accurately diagnosed and treated, patients can avoid the occurrence of life-threatening acute complications to a large extent, such as acute diffuse peritonitis and sepsis.

TOA and OEC appear similar on ultrasound images, but the pathological components other than water in the cystic fluid differ slightly. The main components of TOA are pus, with or without hemorrhage, and granulation tissue formation. Generally speaking, a doctor cannot make a diagnosis merely based on indicators (blood tests, physical exam findings, *etc.*) or US images in a clinical setting. It would be extremely beneficial for the patient if the doctor could comprehensively consider the results of the CNN models and other indicators. Some physicians considered the elevated CA125 concentration as one of the diagnostic indicators for endometriosis. We also analyzed this aspect. The AUROCs, accuracy, sensitivity, and specificity were only 0.564 (0.315–0.813), 0.677 (0.501–0.814), 0.6 (0.313–0.832), and 0.714 (0.5–0.862), respectively. As reported in some studies, plasma CA125 concentrations were significantly increased in some endometriosis patients. However, the CA125 concentration was not raised or slightly raised during the luteal phase in patients with mild endometriosis. In healthy women, the plasma concentration of CA125 was slightly elevated during ovulation and markedly elevated during menstruation. Hence, serum CA125 cannot serve as a typical biomarker for diagnosed endometriosis and has a low value in these clinical situations (Muyldermans et al., 1995).

We deployed three types of CNNs to analyze this task, which have one thing in common: a relatively large number of layers. The greater the number of CNN layers, the more high-level features are obtained after convolution. For this reason, such a model has good performance not only in the classification task of natural images but also in medical images. Deep learning models have many advantages in ultrasound image applications: 1) they can automatically learn useful features from ultrasound images, which can reduce the workload of manual feature engineering. 2) Since deep learning models can learn more complex patterns from data, they can improve analysis accuracy by eliminating physician subjectivity to some extent. 3) Considering that different hospitals may use different ultrasound devices, they can also process images taken by different ultrasound devices, ensuring strong generalization ability of the deep learning model. 4) Deep learning models can perform a diagnosis in a very short time (a few seconds), which can help doctors improve their efficiency and help them diagnose complex diseases. Overall, deep learning has great potential in ultrasound image analysis to provide physicians with more effective tools to improve clinical efficiency and quality. In the image characteristics of ultrasound, TOA is manifested as a complex multilocular cystic mass with thick and irregular cyst walls and septa and mixed and complex echoes inside, and the anatomical boundary between the ovary and fallopian tube is unclear (Chappell and Wiesenfeld, 2012). General sonographic features of OEC are diffuse low-level internal echogenicity, septations, thickened walls, and wall nodularity (Berker and Seval, 2015). To sum up, TOA and OEC have a lot in common on US images, including similar anatomical locations, and both are cystic masses and have wall thickness and septations. Interestingly, in the US images, our model pays more attention to the boundaries of the lesion and the subtle differences in its adjacent organs (Figure 5). Nevertheless, the sonographer is more interested in differences in intralesional composition. However, changes in the internal components are not always consistent at different stages of disease development, which may also be one reason the model performs better. In real time, the differential diagnosis is more than just TOA and OEC. The clinicians will inevitably consider other possible diseases when faced with a diagnosis. However, the advantage of machine learning was that the possible outcomes were only two.

There were some limitations to our study.

1. It was a retrospective study. The results of the model could only illustrate the diagnostic performance of TOA and OEC patients in our institution.
2. Ultrasonography was manual and highly subjective, so different physicians would cause differences in image quality.
3. Our study was single-center, and the performance of the CNN models was considerably high on both internal and independent datasets, which is due to the small test sample size of 11 and 31 patients, respectively.

Hence, we hope to actively cooperate with other centers to carry out multi-center, large-sample prospective studies to further validate these results in larger populations.

In conclusion, we proposed US image-based CNN models that could improve the diagnostic coincidence rate of TOA and OEC. The CNNs could provide a valuable predictive reference for screening TOA and OEC and be combined with other tests to assist physicians in making a diagnosis more precisely.

## Data Availability

The original contributions presented in the study are included in the article/Supplementary Material; further inquiries can be directed to the corresponding author.
